# Editorial: Diet and training strategies to optimize health parameters

**DOI:** 10.3389/fpubh.2025.1556859

**Published:** 2025-02-10

**Authors:** Javier Benítez-Porres, Mora Murri

**Affiliations:** ^1^Physical Education and Sport, Faculty of Medicine, University of Málaga, Málaga, Spain; ^2^Internal Medicine Clinical Management, Málaga, Spain; ^3^CIBER Fisiopatología de la Salud Carlos III, Málaga, Spain; ^4^Endocrinology and Nutrition Clinical Management Málaga y Plataforma en Nanomedicina-IBIMA Plataforma BIONAND, Málaga, Spain; ^5^Cardiology and Cardiovascular Surgery Department, Heart Area, Hospital Universitario Virgen de la Victoria, Málaga, Spain

**Keywords:** nutrition, sport, exercise, lifestyle, body composition, diet, microbiome

Non-communicable diseases (NCDs) represent the leading cause of mortality worldwide, accounting for 43 million deaths annually and 75% of global mortality (1). Over 32 million deaths occur in low- and middle-income countries, with cardiovascular diseases, cancers, chronic respiratory conditions, and diabetes contributing to more than 80% of premature deaths. Physical inactivity and unhealthy diets are significant contributors to the global burden of NCDs.

Nutrition is fundamental for human health, influencing growth, immunity, and disease prevention (2). Adequate nutrition supports maternal and child health, reduces the risk of chronic conditions like diabetes and cardiovascular diseases, and enhances longevity. However, malnutrition, encompassing both undernutrition and overnutrition, remains a global challenge, leading to stunting, nutrient deficiencies, and diet-related chronic diseases (3).

The present Research Topic highlights the interplay between diet, exercise strategies, and lifestyle behaviors in improving health and performance outcomes. By examining physiological and biological mechanisms, the studies explore how combined approaches can optimize health parameters and performance.

## Individual-level factors

Recent changes in dietary patterns have contributed to rising rates of malnutrition and diet-related chronic diseases. Zheng et al. found that healthy low-carbohydrate and low-fat diets reduced the risk of adiposity. Similarly, Zu et al. showed that higher dietary intake of flavonoids significantly reduced weight-adjusted waist index (WWI). Encouraging the consumption of flavonoid-rich foods to reduce obesity and related chronic diseases. These findings underscore the need for population-based interventions to promote healthier diets and reduce disease risk.

## Mental health and nutrition

Mental health also intersects with dietary behaviors in meaningful ways. Jin et al. linked depressive symptoms during pregnancy to impaired intuitive eating behaviors and poorer diet quality, emphasizing the need to integrate mental health support with nutrition education. Tokarek et al. further explored the role of personality traits, finding that neuroticism may led to poorer dietary choices, while conscientiousness was associated with healthier behaviors. Personality-driven interventions, such as stress management workshops and mindfulness-based interventions, can promote resilience and healthier habits in high-stress environments.

## Promising interventions

### Physical activity

Physical activity plays a pivotal role in preventing psychiatric, neurological, metabolic, cardiovascular, pulmonary, and musculoskeletal diseases, as well as cancer (4). Evidence consistently shows that higher levels of physical activity and reduced sedentary behavior lower all-cause mortality, particularly in middle-aged and older adults. Moderate-to-vigorous physical activity also reduces hospitalization risk from conditions like cardiovascular diseases.

Targeted interventions for specific demographics have shown promise. Park and Park found that resistance training significantly reduced hypertension risk, particularly among women, highlighting the need for gender-specific physical activity programs. Similarly, Khatib et al. identified energy availability issues among female athletes in Saudi Arabia, underscoring the necessity of educational programs promoting balanced nutrition and safe training practices.

### Workplace wellness programs

Onofrei et al. found that nurses with chronic conditions experienced higher stress levels, poorer health perception, and higher BMI, along with greater carbohydrate consumption. These findings underline the need for workplace wellness programs addressing mental health, nutrition, and physical activity. Healthcare professionals, often caught in high-stress environments, require targeted interventions to mitigate these risks.

## Environmental and policy considerations

### Urban and socioeconomic factors

Urban environments significantly influence chronic diseases prevalence. Irankhah et al. revealed that improving urban infrastructure and reducing socioeconomic disparities could lower NCD risks. Access to parks, pedestrian-friendly spaces, and affordable nutritious foods fosters healthier communities. Policy makers should prioritize creating environments conducive to healthy living.

Socioeconomic factors also play a pivotal role. Areba et al. identified education and employment as key determinants of food security among pregnant women. The researchers, emphasizing systemic interventions to address structural issues. Policies empowering women through education and economic opportunities can improve household food security and maternal and child health.

### Adherence to health guidelines

Wang et al. demonstrated that adherence to the American Heart Association's Life's Simple 7 (LS7) health guidelines reduced rheumatoid arthritis (RA) risk, particularly among men under 50 and women across all age groups. This highlights the importance of early and consistent lifestyle changes, including maintaining a healthy weight, regular physical activity, and balanced diets. Gender-specific messaging can further enhance public health initiatives.

## Integrated approaches for sustainable outcomes

Integrating dietary and physical activity interventions within broader policy frameworks is essential for sustainable health outcomes. Enhancing food literacy (Zhixue et al.) helps bridge the gap between awareness and action, while global efforts to regulate food marketing and reduce sugar consumption complement local community-specific programs addressing unique cultural and socioeconomic determinants of health.

The interplay between diet, physical activity, mental health, and social determinants forms the foundation of effective interventions. By addressing these interconnected factors, we can create holistic strategies that not only reduce NCD prevalence but also enhance overall quality of life.

Addressing NCDs requires a dual approach: tackling global challenges while tailoring interventions to local contexts. Globally, collaborative efforts are needed to regulate food marketing, reduce sugar consumption, and promote physical activity through international campaigns. Locally, community-specific programs can address unique cultural, economic, and social determinants of health.

## Future directions

Future research should focus on evaluating the long-term impact of combined dietary and physical activity interventions. Policymakers, healthcare professionals, and community leaders must collaborate to implement evidence-based solutions, ensuring accessibility and sustainability. Together, these efforts can pave the way for healthier individuals, communities, and nations.
